# Thin Glass Micro Force Plate Supported by Planar Spiral Springs for Measuring Minute Forces

**DOI:** 10.3390/mi14051056

**Published:** 2023-05-16

**Authors:** Taisei Kiriyama, Kenichiro Shimazaki, Rihachiro Nakashima, Hidetoshi Takahashi

**Affiliations:** Department of Mechanical Engineering, Faculty of Science and Technology, Keio University, Yokohama 223-8522, Japan; lupin3.1212@keio.jp (T.K.); shimaken1129@keio.jp (K.S.); na8.3436@keio.jp (R.N.)

**Keywords:** force plate, laser processing, thin glass

## Abstract

Microforce plates are indispensable tools for quantitatively evaluating the behavior of small objects such as tiny insects or microdroplets. The two main measurement principles for microforce plates are: the formation of strain gauges on the beam that supports the plate and the measurement of the deformation of the plate using an external displacement meter. The latter method is characterized by its ease of fabrication and durability as strain concentration is not required. To enhance the sensitivity of the latter type of force plates with a planar structure, thinner plates are generally desired. However, brittle material force plates that are both thin and large and can be fabricated easily have not yet been developed. In this study, a force plate consisting of a thin glass plate with a planar spiral spring structure and a laser displacement meter placed under the plate center is proposed. The plate deforms downward when a force is exerted vertically on its surface, resulting in the determination of the applied force using Hooke’s law. The force plate structure is easily fabricated by laser processing combined with the microelectromechanical system (MEMS) process. The fabricated force plate has a radius and thickness of 10 mm and 25 µm, respectively, with four supporting spiral beams of sub-millimeter width. A fabricated force plate featuring a sub-N/m spring constant achieves a resolution of approximately 0.01 µN.

## 1. Introduction

In recent years, sensors capable of measuring with a high resolution of μN or less have been extensively developed [1,2,3,4]. One of the motivations for developing such sensors is the observation of the behavior of living cells and tiny forces generated by small animals [5,6]. Among these sensors, micro force plates are widely used as powerful tools for measuring insect ground reaction force (GRF) or microdroplet collision force [7,8,9,10,11].

Conventionally, force plates have resistors formed at the base of the beam supporting the plate [7,8,9,10,11,12,13,14,15,16]. The force exerted on the plate surface is determined by measuring the change in resistance caused by the applied force. Although this type of force plate is sufficiently sensitive, its development requires a complex microelectromechanical system (MEMS) process or minute and complex manual assembly, and a fragile structure owing to the concentrated strain. Another measurement principle configures the system as a force plate using a mechanical structure and external displacement meter [17,18,19]. Although the measurement system is complex, the force plate element has no electrical components, which simplifies the fabrication process. In addition, the force plate can be constructed with an unbreakable structure because strain concentration is not required. Methods for optically measuring force using a gel plate have been developed. However, their accuracy is concerning [20,21].

When developing a single-axis force plate with a vertical target axis, one of the easiest ways is to fabricate the device in-plane. In particular, for force plates in which the sensor structure and displacement meter are separated, a uniform planar structure is sufficient because strain concentration is not necessary in the out-of-plane direction. To improve the sensitivity of force plates with planar structures, they must be made thinner, thus resulting in a small aspect ratio. However, force plates composed of brittle materials, with a thickness of tens of microns and plate area of tens of millimeters, that can be easily fabricated have not yet been developed.

Thus, the proposed force plate comprises a 25-µm-thick glass plate with a planar spring structure. A laser displacement meter was positioned beneath it to measure the vertical displacement at the center of the plate. A planar spring structure can be easily fabricated using laser processing. Recently, laser processing has been widely used in microfabrication [22,23]. A previous study developed a force plate using a 100-µm-thick polyimide film [18], and the device achieved a resolution of less than 0.02 µN, thereby demonstrating its suitability for measuring the body weight and mass of each body part of a fruit fly. However, despite its easy handling and unbreakable characteristics, polyimide is unsuitable for long-length measurements owing to its viscoelastic properties, which cause gradual deformation after a large load is applied. This causes difficulties, such as requiring a long time to return to the zero point and easily shifting to the zero point.

In this study, glass was selected as the material, and a spiral spring structure was fabricated using ultraviolet (UV) laser processing after marking an aluminum pattern using vapor deposition. The mechanical properties of the fabricated devices are evaluated.

## 2. Design, Principle, and Development

### 2.1. Design and Principle

**Figure 1:**
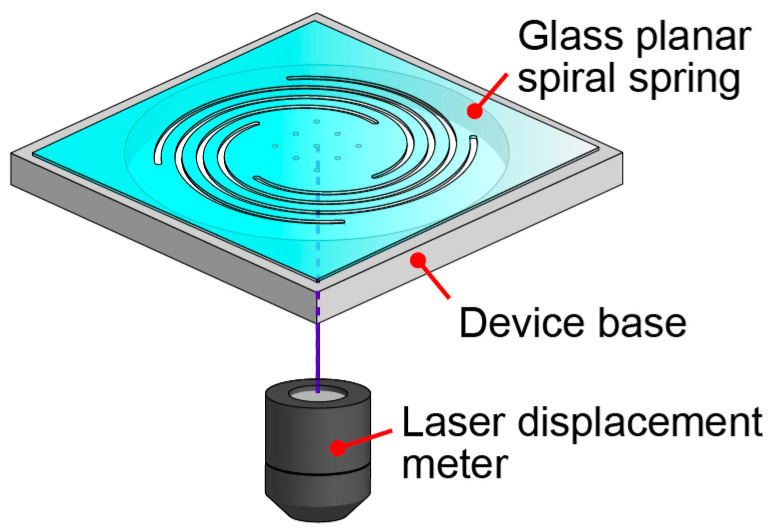
Conceptual diagram of the proposed thin glass micro force plate.

Figure 1 shows a conceptual diagram of the proposed glass force plate. The developed force plate comprised a planar spiral spring structure with four spirals extending from a circular plate at the center. A laser displacement meter was installed under the plate. Each spiral spring had the same shape as that of an Archimedean spiral. The four springs were evenly placed at 90° intervals and did not affect each other. The glass plate contained a pattern to align the measurement point of the laser displacement meter at the center of the plate.

When a vertical force was applied to the plate surface, the spirals were deflected, thereby causing a vertical plate displacement, which was detected using a laser displacement meter. Here, let the applied force be *F*, the vertical displacement of the plate center be *Z*, and the pseudo-spring constant of each spiral spring be *k*; then, Hooke’s law is as follows.

(1) F=4kZ.

Therefore, *F* is calculated using the premeasured spring constant and measured displacement. If the four springs are mechanically identical, the applied force can be calculated solely by the total spring constant and center displacement, regardless of the position of the applied force [17]. Positional errors may arise if variations exist among the four springs or if the measurement point deviates from the center. Thus, precise fabrication and alignment are important.

The spring constant determines the sensitivity of the force plate. The spiral shape of the four springs supporting the central plate allows for a high concentration of spiral springs capable of generating significant displacements. Furthermore, altering the parameters of the spiral shape is relatively simple. The spring constant depends on the length, width, and thickness of the spiral. In particular, it is proportional to the cube of the thickness; this implies that it reduces significantly as the glass becomes thinner, thereby resulting in greater sensitivity.

### 2.2. Plate Design

To develop a force plate suitable for measuring tiny insects, such as fruit flies, the required dimensions, force resolution, and resonant frequency were determined, similarly to in a previous study [18]. The body of a fruit fly is approximately 3-mm-long, and a plate diameter of 10 mm was assigned to ensure that the plate was sufficiently large to accommodate a fruit fly. The resolution of the laser displacement meter was 10 nm and the weight of the fruit fly’s body was approximately 1 mg. Herein, the force resolution was set to 1 N/m or less to obtain sufficient resolution for weight measurement. As the frequency of a fruit fly’s gait is approximately 20 Hz during walking [24], the resonant frequency was set to 25 Hz or higher.

**Figure 2:**
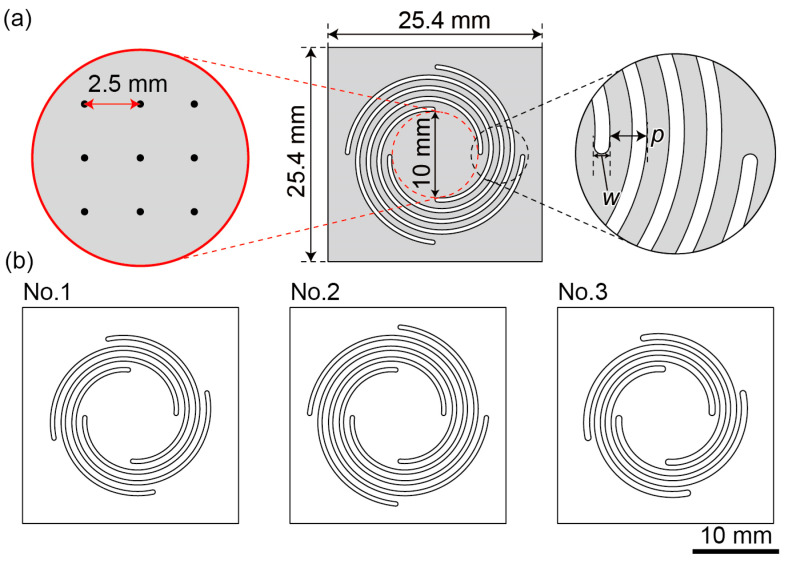
(**a**) Detailed design of the three types of force plates. (**b**) Each force plate design according to the parameters presented in Table 1. The spring width *w*, pitch *p*, and turn *N* differ in each case.

A schematic of the proposed planar spiral spring is shown in Figure 2a, and the parameters for each of the three spirals are listed in Table 1. The device has a square of side 25.4 mm with a plate of diameter of 10 mm at the center, from which four spiral shapes were removed. In this study, the pitch *p* of each spiral was 1.25 mm, and the mechanical properties, such as the spring constant and resonant frequency, were adjusted by varying the cutting width *w* and the number of turns *N*. The arrangement of the nine points such that they accurately determine the center of the plate and assess corner-weighing differences is shown in Figure 2a. To avoid stress concentration, a rounded shape with the same diameter *w* was formed at the beginning and end of the spiral.

Every spiral shares a shape identical to that of the Archimedes spiral. Herein, the radius at any given point on the spiral, denoted as *r* [mm], and the central angle, denoted as *θ* [rad], are defined as follows. When the cutting width *w* and number of turns *N* are determined, the relationship between *r* and *θ* is expressed by Equations (2) and (3):

(2) $$r=5\mp \frac{w}{2}+\frac{5\theta }{8\pi }(0\leq \theta \leq \theta_{\max}),$$

(3) $$\theta_{max}=N\times 2\pi$$

In this study, a spring with the three parameters listed in Table 1 was used. Each design drawing is shown in Figure 2b. The thickness of the glass wafer used in the experiments ranged between 22 and 28 µm. For the simulation, described subsequently, an average thickness of 25 µm for 10 wafer samples was utilized.

**Figure 3:**
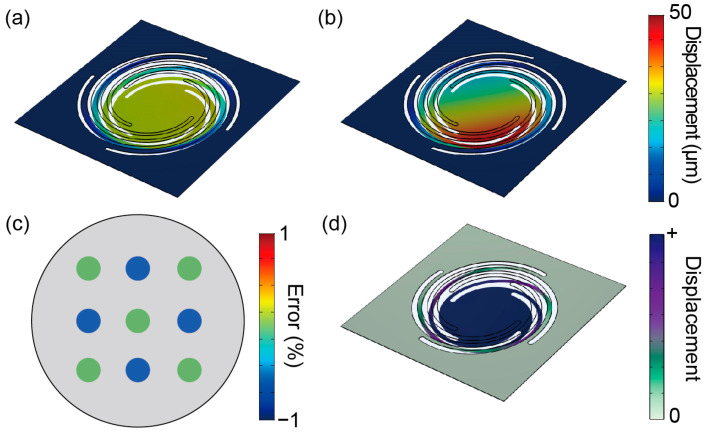
Simulation outcomes depicting the deformation resulting from the application of a force to (**a**) the plate center and (**b**) to the plate edge in design No. 2. (**c**) Spring constant error distribution of plate No. 1 in the simulation. (**d**) Mode shape at the first resonant frequency.

The resonant frequencies and spring constants for each force plate were calculated using the finite element method (COMSOL Multiphysics 5.6, COMSOL, Burlington, VT, USA). Herein, borosilicate glass with a density of 2.2 g/cm^3^, Young’s modulus of 72 GPa, and Poisson’s ratio of 0.23 was used as the device material. All four sides of the force plate were fixed and a vertical force of 10 µN was applied to it. Figure 3a shows the deformations of plate No. 2 when a vertical force was applied to its center; the spring constant was calculated as 0.231 N/m. Figure 3b shows the deformation when a force was applied to the plate edge; in this case, the displacement varied according to the distance from the location where the force was applied. However, the central displacement was almost constant, regardless of the position of the applied force. Figure 3c shows the positional error of the spring constant of the plate (No. 1) when a force was applied at the nine points mentioned above compared with the spring constant when a force was applied at the center. The error was within 1%, which is sufficiently small. Similarly, small positional errors were obtained for the other two force plates. Figure 3d shows the mode shape of plate No. 3 at the first resonant frequency. The plate was confirmed to be in the vertical up/down mode. Table 2 summarizes the simulation values of the resonant frequencies and spring constants. Evidently, all resonant frequencies were above 25 Hz, and No. 1 had the highest resonant frequency. Further, the spring constants were all less than 0.5 N/m, and No. 2 was the most sensitive force plate.

### 2.3. Fabrication

**Figure 4:**
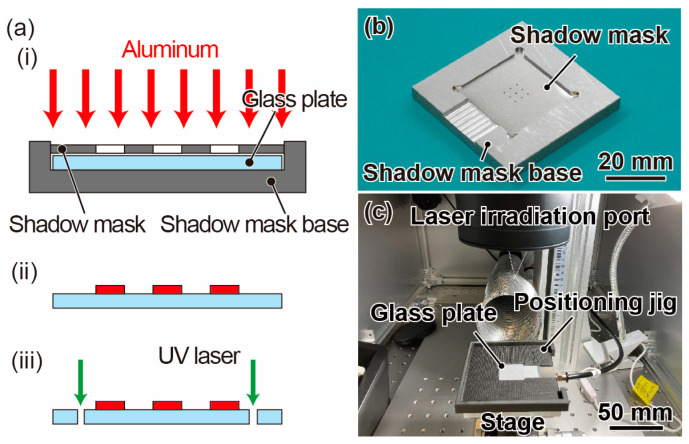
(**a**) Fabrication of the force plate. (**i**) Aluminum deposition using the shadow mask. (**ii**) After aluminum deposition. (**iii**) Cutting the glass wafer with UV laser processing machine. Photographs of the (**b**) shadow mask system and (**c**) alignment of the UV laser machine.

Figure 4a shows the fabrication process of the proposed force plate. The starting material was a 1-inch glass wafer with a thickness of approximately 25 µm (Glass Ribbon, Nippon Electric Glass Co., Ltd., Otsu, Japan). First, the glass wafer was placed on top of a shadow mask base, and a shadow mask with nine holes was placed on the glass plate, as shown in Figure 4b. The shadow mask was fabricated from a 75 µm thick aluminum plate using a fiber laser processing machine (FIBER-PROF-001, Profitet Co., Ltd., Saitama, Japan); the radius of the hole was 0.4 mm. The shadow mask base was designed to precisely align the glass plate and shadow mask in the center, thus enabling the accurate positioning of the nine holes at the center of the glass plate. This setup enabled aluminum deposition in the holes. The deposition target was placed at the bottom of the deposition apparatus (VE-2012, Vacuum Device Co., Ltd., Mito, Japan), whereas the deposition source aluminum was positioned at the top. The thickness of the deposited aluminum was of the order of 100 nm, which did not affect the mechanical properties. After aluminum deposition, the glass wafer was cut using a UV laser processing machine (UV-PROF-001, Profitet Co., Ltd.). The process parameters are listed in Table 3. A positioning housing fabricated using a 3D printer (Onyx One, Markforged, Watertown, MA, USA) was used to center the glass wafer when it was placed on the processing stage. This enables the fabrication of a spring structure with symmetrical top-to-bottom and left-to-right orientations without biasing the portion, as shown in Figure 4c.

**Figure 5:**
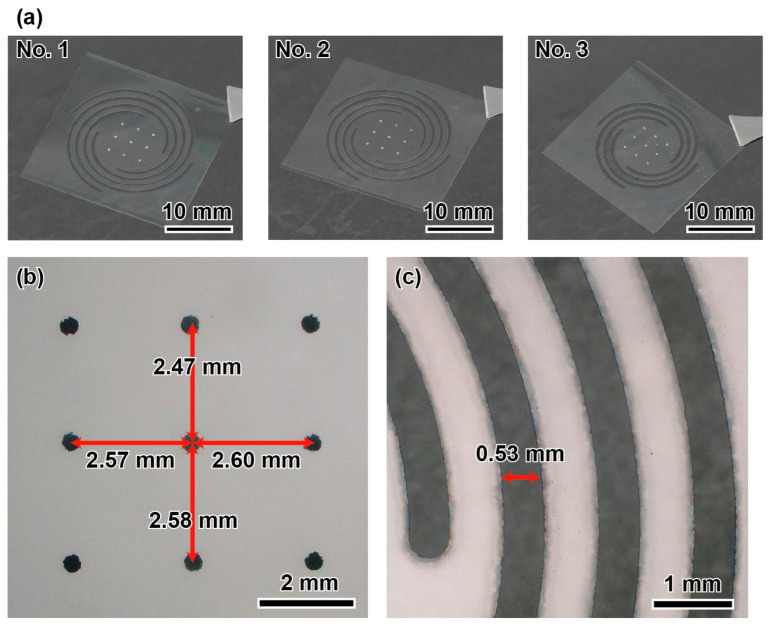
Photographs of the (**a**) fabricated force plates according to the parameters and designs (Nos. 1–3) shown in Figure 2, (**b**) plate center, and (**c**) spirals.

**Figure 6:**
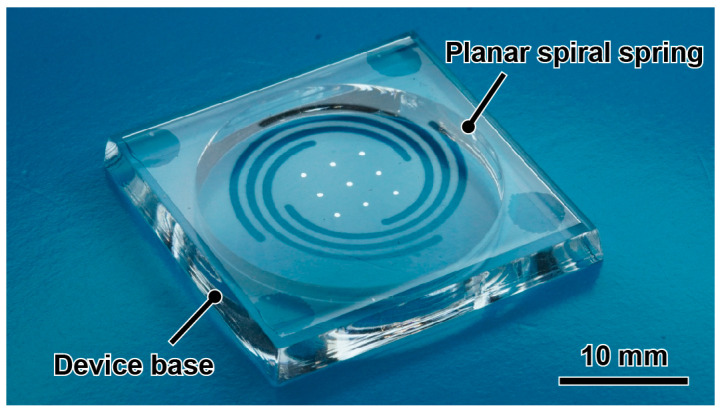
Photograph of the assembled device.

Figure 5a shows the fabricated force plates according to the parameters and designs (Nos. 1–3) shown in Figure 2, respectively, whereas Figure 5b,c shows the close-up images focusing on the plate center and spiral, respectively. The sides of the UV laser-irradiated glass had no cracks. Regarding the deposition process, nine points were first deposited as easily visible dots. The distance between each point was 2.47–2.60 mm, which is within a 5% error range compared with the designed distance of 2.5 mm. Compared with previous landmarks on the force plate, such as the grid lines of the MEMS force plate array [7], the error range was large, owing to the shadow-mask process. However, there was little influence on the calibration. The lengths from the center point of the aluminum deposition to the spiral end points were distributed in the range of 4.82–5.23 mm, which is also within a 5% error range. Possible sources of error include the dimensional error of the shadow mask during deposition and errors in the installation of the glass plate during laser processing. In addition, the cutting width *w* was approximately 30 µm larger than the designed value; this error occurred owing to the spot diameter of the UV laser. Figure 6 shows a photograph of the force plate attached to a device base fabricated by machining an acrylic plate of a height of 3 mm.

**Figure 7:**
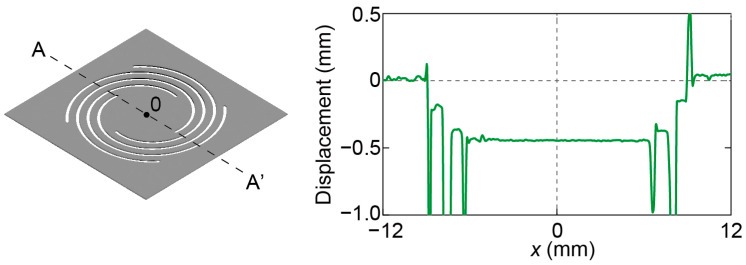
Initial displacement of the entire force plate.

The displacement of the fabricated device with plate No. 1 under its own weight measured by a laser microscope (VR-5000, Keyence Corporation, Osaka, Japan) is shown in Figure 7. The plate initially sank approximately 0.5 mm in the center. This displacement is reasonable considering the plate weight and designed spring constant.

## 3. Experiments and Results

### 3.1. Resonant Frequency

**Figure 8:**
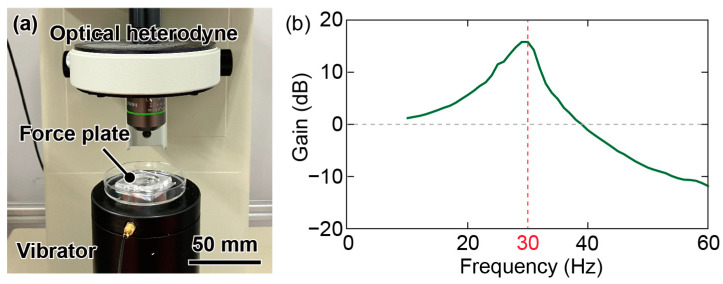
(**a**) Photograph of the experimental setup for evaluating the resonant frequency. (**b**) Frequency response of force plate No. 1.

The experimental setup used to evaluate the resonant frequency is illustrated in Figure 8a. The fabricated device was attached to a vibrator (Mini-shaker Type 4810, Brüel and Kjær, Virum, Denmark) and vibrated vertically from 10 to 100 Hz in increments of 1 Hz. The amplitudes of the vibrations were measured using a laser heterodyne vibrometer (MLD-221 D-DWT, NEOARK, Hachioji, Japan) and analyzed using a frequency analyzer (FRA5087, NF Corporation, Yokohama, Japan). Measurements were conducted at the plate center and fixed jig. The difference between the two outputs is shown in Figure 8b. The first resonant frequency of plate No. 1 was approximately 30 Hz, whereas those of plates No. 2 and No. 3 were 25 and 27 Hz, respectively. All the experimental values were approximately 20% smaller than the simulated values. This difference may have arisen because the spiral width is narrower than that in the design based on the spot diameter of the laser beam. In addition, the glass wafer used in the fabrication may have been thinner than that used in the simulations.

### 3.2. Experimental Setup

**Figure 9:**
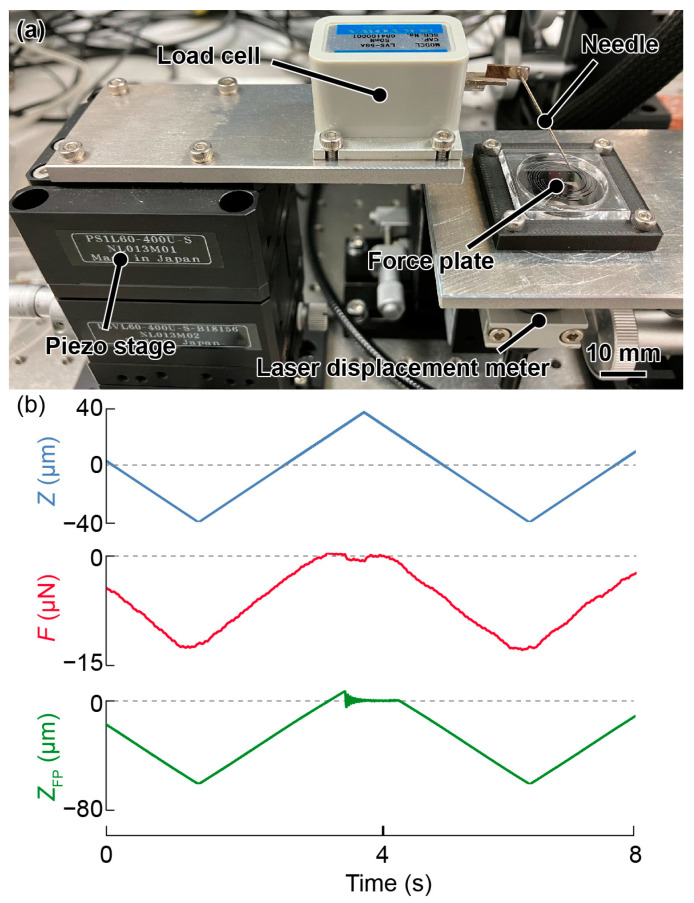
(**a**) Photograph of the experimental setup to evaluate of sensitivity variation. (**b**) Measured values of *Z*, *F*, and *Z*_FP_ obtained while pushing the needle tip at the center.

The experimental setup to evaluate the responses to the force is shown in Figure 9a. A load cell (LVS-5GA, Kyowa Electronic Instruments Co., Ltd., Chofu, Japan) was placed on a piezo stage (PSVL-400U-S, THK PRECISION Co., Ltd., Tokyo, Japan) and a needle was placed on the load cell. A multicolor confocal laser displacement meter (CL-L015 and CL-3050, Keyence Corporation, Osaka, Japan) was placed under the plate to detect the displacement at the plate center. The laser displacement meter was almost independent of temperature (0.005% F.S./°C). In principle, any type of external laser displacement meter, such as an interferometer or triangulation method, can be used.

A triangular wave was input to the piezo stage from a function generator (WF1974, NF Corporation), which induced vertical oscillations of the piezo stage. Subsequently, the needle moved vertically so that the force was applied via the needle tip. The displacement of the piezo stage, displacement detected by the laser displacement meter, and applied force measured by the load cell were simultaneously recorded on a scope coder (DL850E, Yokogawa Test & Measurement Corporation, Hachiōji, Japan). The influence of the needle deformation on the measurement results was negligible as the spring constant of the needle was sufficiently large. Herein, a displacement of approximately 50 µm was applied at 0.2 Hz.

### 3.3. Sensitivity Evaluation

**Figure 10:**
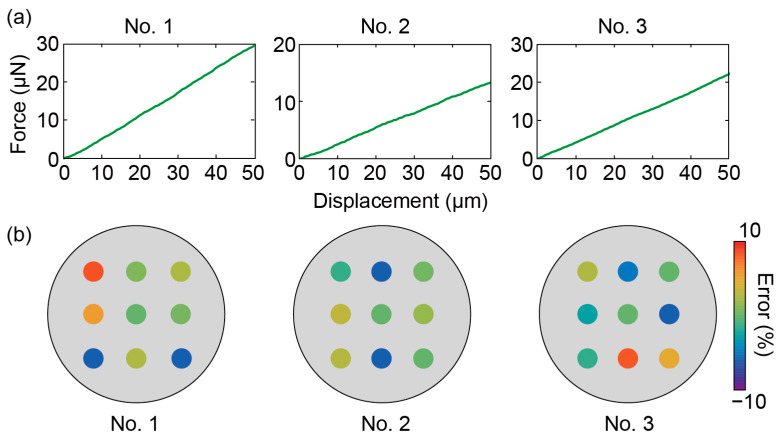
(**a**) Relationship between the applied force *F* and *Z*_FP_ at each point. (**b**) Sensitivity variation obtained experimentally.

The displacement *Z* of the piezo stage, force *F* from the load cell, and force plate output *Z*_FP_ were recorded as shown in Figure 9b. The sampling rate and applied low-pass frequency were 500 and 40 Hz, respectively. At approximately 3.5 s, *F* and *Z*_FP_ were zero when the needle left the plate’s surface. The plots in Figure 10a show the displacement on the horizontal axis and the force on the vertical axis for the three different force plates, namely, plates 1–3. The spring constant was determined by calculating the slope of the fitted line on the graph.

The position error distribution of the spring constant was experimentally evaluated by pushing the nine points of the aluminum pattern. The experimental position error in the spring constant is shown in Figure 10b. The position error was within ±10% for each plate. Unlike in the simulation, no regularity was observed in the distribution, and it was slightly larger than that in the simulation. A summary of the experimental results is provided in Table 4.

The laser displacement meter had a resolution of 10 nm, and the theoretical resolution was obtained by multiplying this value by the spring constant of the spiral spring. However, because the device was much thinner, small vibrations during the experiment were expected to cause mechanical noise in the measurement. Therefore, the displacement without force was recorded using a laser displacement meter to evaluate the noise level. The experiment was conducted for 60 s while a cover was attached to the device to block the influence of air. Next, the standard deviation was obtained as 0.02–0.04 µm. The force resolution was defined by multiplying each of these values by the spring constant, which was less than 0.01 µN and was sufficiently high compared with those of previous force plates.

## 4. Discussion

Compared with previous polyimide force plates of the same size and with a similar spiral structure [18], the fabricated glass force plate had a higher resonant frequency, although the force resolution was the same. This is because the glass wafer was thinner than the polyimide film (100 μm), thereby resulting in a lighter weight. In addition, aluminum vapor deposition was used to deposit the markers. Photolithography is also a feasible technique instead of shadow masks for a glass wafer of such thicknesses. Similar to previous spiral force plates with external displacement meters [18,19], the fabricated force plate was easy to handle and unbreakable. However, at different points from the polyimide force plate, the initial displacement was small and there was no gradual deformation.

The proposed device was fabricated using a simple fabrication process. Devices with different mechanical properties can be easily fabricated by changing the parameters of the spring structure. In terms of material selection, glass is extremely suitable for manufacturing planar force plates with a size of 10 mm and a spring constant of 1 N/m. Although a conventional polyimide film can also be easily processed, similarly to glass, it is extremely soft, with a thickness of approximately 10 μm, and may deform plastically under its own weight to a greater degree. An attempt to process the same dimensions in a metal material is likely to cause plastic deformation. Anisotropic materials, such as silicon, are also likely to break during processing at such thicknesses. In terms of price, glass wafers have an advantage over silicon wafers for such ultrathin films. In this study, glass wafers with a thickness of 20 μm were used; however, in principle, wafers of 10 μm or less can also be fabricated. In this case, the force resolution is expected to increase by a factor of approximately 10 if the shape remains the same. Compared to conventional resistive force plates, the initial cost of a laser displacement meter is required. However, the fabrication cost of the plate itself is sufficiently low. Therefore, it is feasible to commercialize the proposed force plate system using a suitable plate according to the measured object by preparing multiple plates in advance.

## 5. Conclusions

In this study, a thin glass microforce plate with planar spiral springs was fabricated and evaluated. When a target was positioned on the plate surface, the laser displacement meter located beneath the plate detected the vertical displacement of the plate center. This enabled the calculation of the force based on the displacement using Hooke’s law. The force plate structure was fabricated by aluminum deposition with a shadow mask and laser processing, which is an easier process compared with utilizing conventional force plates and using MEMS processes. The experimental results indicated a resonant frequency greater than 25 Hz and a spring constant less than 0.01 μN. Therefore, the proposed force plate is expected to be a powerful tool for measuring minute forces.

Using thinner glass allows for improvements in both sensitivity and resonant frequency. However, it should be noted that extremely thin glass can increase costs and be more challenging to handle. Therefore, our device has one of the most optimal designs for a plate size of 10 mm in diameter. If higher sensitivity is desired, a more robust system, such as housing to construct the plate and laser displacement meter, would be advisable. Furthermore, the sensitivity of the force plate depends on the performance of the laser displacement meter. Therefore, if higher resolution laser displacement meters become commercially available in the future, the sensitivity of force plates can be further improved.

## Figures and Tables

**Table 1:** Parameters of the three types of force plates.

	*w* (mm)	*p* (mm)	*N* (—)
No. 1	0.5	1.25	1.6
No. 2	0.5	1.25	2.0
No. 3	0.7	1.25	1.6

**Table 2:** Mechanical properties of the three types of force plates in simulation.

	Resonant Frequency (Hz)	Spring Constant (N/m)	Position Error (%)
No. 1	39.5	0.460	0.62
No. 2	26.6	0.231	0.92
No. 3	34.2	0.326	0.87

**Table 3:** Parameters of the UV laser processing machine.

Mark Loop	Speed	Power	Frequency	Point Dist.
160	5000 mm/s	100%	10 kHz	0.5 mm

**Table 4:** Experimental results of the three types of force plates.

	Resonant Frequency (Hz)	Spring Constant (N/m)	Position Error (%)
No. 1	30	0.319	8.5
No. 2	25	0.224	6.0
No. 3	27	0.260	8.3

## Data Availability

The data presented in this study are available upon request from the corresponding author.
